# Automated Large-Scale Extraction of Nanotoxicity Data from the Literature Using Multimodal Large Language Models

**DOI:** 10.34133/csbj.0161

**Published:** 2026-07-07

**Authors:** Seung-Geun Park, Seung Min Ha, Zayakhuu Gerelkhuu, Tae Hyun Yoon

**Affiliations:** ^1^ Department of Chemistry, Hanyang University, Seoul 04763, Republic of Korea.; ^2^ Research Institute for Convergence of Basic Science, Hanyang University, Seoul 04763, Republic of Korea.; ^3^ Institute of Next Generation Material Design, Hanyang University, Seoul 04763, Republic of Korea.; ^4^ Yoon Idea Lab. Co., Ltd. Seoul 04763, Republic of Korea.

## Abstract

Manual curation of nanotoxicity data remains a major bottleneck in the scalable construction of large datasets because key physicochemical and toxicological variables are often distributed across text, figures, tables, captions, legends, and Materials and Methods sections. Here, we developed a multimodal large language model (LLM) workflow for automated extraction and structured curation of nanotoxicity data from research articles. Four multimodal LLMs were benchmarked on curated set of 17 oxide nanotoxicity articles. GPT-5.4 provided the best balance between extraction accuracy and efficiency, achieving a mean extraction F1 score of 96.1% and an assay results F1 score of 86.5% on the main benchmark. The optimized workflow was then applied to 821 articles, generating 16,786 structured records and substantially reducing the manual recovery of figure-derived quantitative data. Automated machine learning (AutoML) modeling showed that the physicochemical (PChem) score-filtered subset achieved higher overall predictive performance, whereas record-count balancing among the 4 most represented materials reduced performance. Models trained only on records for ZnO, TiO_2_, Fe_3_O_4_, or SiO_2_ improved prediction for the corresponding material compared with multi-material models, but applicability domain analysis showed that these gains occurred within narrower descriptor-space coverage. These findings demonstrate that multimodal extraction can move nanotoxicity curation beyond text-only workflows while supporting data-aware model selection across broad-coverage, quality-filtered, focused multi-material, and single-material prediction strategies.

## Introduction

With the increasing use of nanomaterials (NMs), robust and reliable assessment of their potential toxicity has become essential for nanosafety evaluation and safer material design [1–3]. Data-driven approaches, including nanostructure–activity relationship (nanoSAR) analysis and machine-learning models, offer a scalable complement to conventional in vitro and in vivo testing strategies [4–8]. Their usefulness, however, critically depends on the availability of large, well-structured, and well-curated datasets [7,9–12]. In practice, assembling such datasets remains a major bottleneck because physicochemical properties and toxicological outcomes must often be manually curated from published studies [9–12].

In our previous work, we developed a large language model (LLM)-based workflow for extracting nanotoxicity data from oxide NM (OxideNM) literature and showed that text-based extraction could support downstream predictive modeling [13]. That framework successfully recovered material identities, physicochemical descriptors, and several toxicological metadata fields. However, important quantitative assay variables remained difficult to extract automatically, particularly when they were presented in figures rather than in plain text. In that workflow, exposure dose, exposure time, and cell viability had to be manually recovered from graphical data, and similar limitations could also affect physicochemical variables such as hydrodynamic size and surface charge when those values were reported outside the main text [13–15]. Thus, although the previous workflow improved extraction efficiency, it did not fully eliminate the manual steps that limit the scalability of nanotoxicity data curation.

This limitation is especially important in nanotoxicology because the information needed to reconstruct complete physicochemical and toxicological records is inherently multimodal, being distributed across figures, tables, captions, legends, and Materials and Methods sections rather than stated explicitly in the main text [14–16]. As a result, text-only approaches can recover contextual descriptors but often fail to capture the quantitative values required for a modeling-ready dataset. Recent advances in optical character recognition, computer vision, and multimodal document analysis offer a potential route to address this limitation, yet their application to nanotoxicity literature remains limited [12,16–19].

In the present study, we developed a multimodal extraction workflow for automated extraction of OxideNM data from the literature. Rather than relying solely on text retrieval, the workflow integrates textual and visual information from full articles to assemble structured physicochemical and toxicological records. It was designed to recover quantitative assay variables such as exposure dose, exposure time, and assay response while also extending extraction to physicochemical values reported in tables or characterization figures. Importantly, the main contribution of this study is not only the use of multimodal models but also the development of a reproducible framework for automated nanotoxicity data extraction and structured curation.

To maintain continuity with our previous work, we focused on OxideNMs and in vitro cytotoxicity literature and applied the same overall data quality control framework [13]. We benchmarked 4 multimodal LLMs and evaluated both extraction accuracy and practical efficiency using a curated oxide literature dataset. The selected workflow was then applied to a larger oxide literature collection to generate multimodally extracted, structured datasets for downstream AutoML-based nanotoxicity prediction. In addition, as an exploratory application, we examined whether the resulting datasets could support both broad oxide-wide modeling and narrower material-specific prediction strategies. Overall, this study evaluated whether multimodal extraction can reduce key manual bottlenecks in data curation and enable scalable, structured dataset construction while also clarifying how dataset scope affects both material-specific prediction performance and applicability domain (AD) coverage.

## Materials and Methods

### Overall study design and workflow

This study was conducted in 3 stages: (a) multimodal extraction of physicochemical and toxicological data from OxideNM literature, (b) benchmark evaluation of extraction performance across multiple LLMs, and (c) predictive modeling of nanotoxicity using AutoML platforms and the extracted datasets. The overall workflow is shown in Fig. 1. Compared with our previous text-based approach, the present workflow expands the extraction scope to include quantitative values reported in figures and tables, together with their associated textual context. Accordingly, the workflow targeted both physicochemical descriptors, including hydrodynamic size and surface charge, and toxicological assay variables, including exposure dose, exposure time, and assay response. These extracted data were subsequently used for model benchmarking and downstream predictive analysis.

**Fig. 1.**
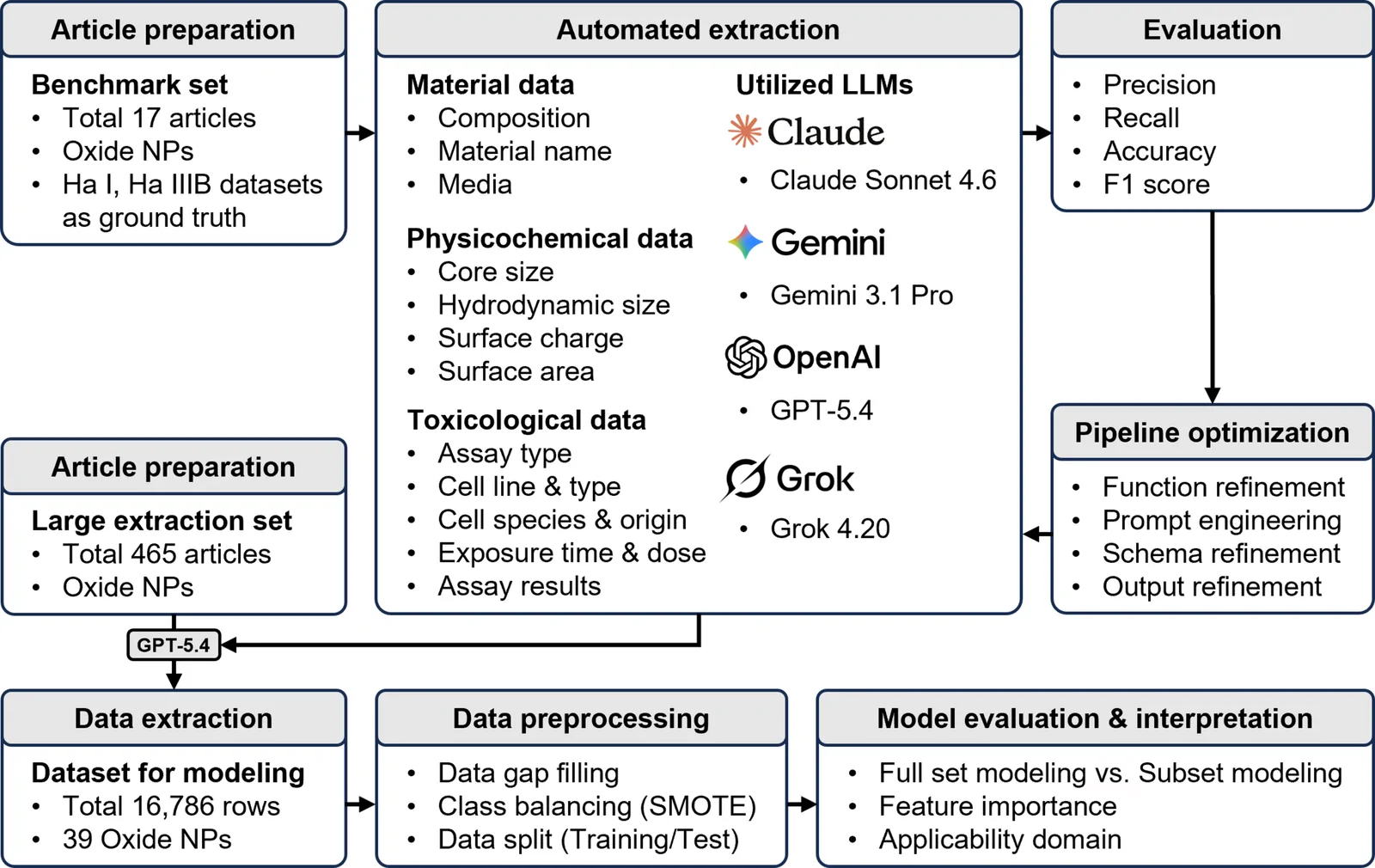
Overall workflow of the proposed framework.

### Literature sources and dataset design

This study focused on OxideNM literature reporting in vitro cytotoxicity assays. Doped materials were excluded, whereas composite OxideNMs were included. Following the dataset-construction framework established in the previous study [20]—where the detailed multi-expert curation, verification, and data reconciliation protocols are fully documented—3 reference datasets were used. Ha I represents the original dataset extracted from all 216 papers prior to data gap filling and quality filtering. Ha II was generated from Ha I after data gap filling but before PChem score-based quality screening. During workflow development, the prompt/schema refinement process used to finalize the fixed PChem and Tox extraction prompts, output fields, and postprocessing rules before model benchmarking is summarized in Fig. S1. Ha IIIB corresponds to the PChem score-filtered subset derived from Ha II by selecting records from papers within the top 20% of papers ranked by PChem score, comprising 17 articles. Ha I and Ha IIIB were used as ground truth reference datasets for extraction-pipeline development and benchmarking.

After LLM benchmarking and model selection, the optimized multimodal workflow was applied to oxide literature pool of 821 articles assembled in our previous study [13,20]. Following automated extraction, articles were retained only if the extracted records corresponded to in vitro cytotoxicity assays involving OxideNMs, yielding 465 articles. The resulting dataset, Park-OxideNM2026, served as the broad-coverage dataset for downstream predictive modeling.

### Multimodal data extraction pipeline

The multimodal extraction pipeline was implemented in Python using a LangChain-based orchestration framework [21]. To enable controlled comparison across models, all pipeline components, including portable document format (PDF) retrieval, page parsing, prompt design, structured output schema, multimodal input construction, and postprocessing, were kept constant, and only the LLM provider varied. The evaluated LLMs were Claude Sonnet 4.6 (claude-sonnet-4-6), Gemini 3.1 Pro (gemini-3.1-pro-preview), GPT-5.4 (gpt-5.4-2026-03-05), and Grok 4.20 (grok-4.20-0309-reasoning). For all LLM calls, the temperature was set to 0 to reduce stochastic variation, and all other generation parameters were left at the default settings provided by each model provider unless otherwise required by the application programming interface (API). Article PDFs were retrieved programmatically from a Zotero group library through the pyzotero API [22]. For each selected collection, the pipeline enumerated parent records, inspected child attachments, retained records with a single PDF attachment, and downloaded uncached files by attachment key. Full-paper text was extracted page by page using PyMuPDF and preserved with explicit page markers. A 2-stage cleaning procedure was then applied. First, deterministic normalization corrected common PDF artifacts, including null (NUL) characters, soft hyphens, ligatures, dash variants, and inconsistent unit glyphs. Second, only heuristically flagged spans were subjected to minimal LLM-based repair under a strict JavaScript Object Notation (JSON) schema to correct corrupted text without paraphrasing or altering numeric values. The Materials and Methods section was then identified from the repaired page-preserving text using rule-based heading patterns.

NMs used in biological assays were extracted from the full-paper text rather than from local text chunks. One record was created for each material-medium condition after normalization of exposure-medium names. Figures and tables were extracted from PDFs by rasterizing each page and detecting layout regions with LayoutParser using a PubLayNet-based Mask R-CNN model [23,24]. Detected regions were cropped, and captions were reconstructed from PyMuPDF text blocks by merging adjacent caption fragments and assigning the nearest caption of the corresponding object type. Cropped figure and table images were converted to RGB JPEG format, resized when necessary, and encoded for multimodal input. In this workflow, material (Mat) extraction was primarily text-based, whereas physicochemical (PChem) and toxicological (Tox) extraction integrated both textual and visual information, including figures, tables, and captions.

For PChem extraction, captions were first screened to identify candidate figures and tables likely to contain characterization data for each target material. Candidate values for core size, hydrodynamic size, surface charge, and surface area were then extracted using material metadata, nearby page text, relevant full-text context, captions, and cropped images or tables. Each candidate value was stored with provenance fields, including the raw source expression, page number, candidate identifier, source type, source origin, and evidence text. Candidate values were consolidated into final material-level records using property-specific scoring rules that prioritized usable values, experimentally grounded evidence, relevant medium context, and recognized measurement methods, with unit normalization to canonical forms. A second extraction pass was performed when properties remained unresolved.

For Tox extraction, target material-medium rows were generated from the curated PChem outputs. Candidate toxicity values were extracted by integrating text, captions, and visual inputs, while the Materials and Methods section was used only for contextual validation. Importantly, numeric toxicity values were accepted only when directly supported by local evidence. Extracted values were then standardized through assay normalization, cell line harmonization, and conversion to a common 0 to 100 viability scale. Only records satisfying consistency and completeness criteria were retained. For toxicity records, source provenance was retained at the row level to support contextual verification. Each extracted toxicity claim stored the source page, source type, source caption, panel label, *x*-axis title, *y*-axis title, endpoint type, value origin, and evidence text where available. For figures containing multiple dose–response curves or multiple series, the extraction procedure required inspection of panel labels, legends, line labels, color labels, marker labels, bar-pattern labels, and group labels before assigning a datapoint to a specific material–cell–assay–time–dose condition. A toxicity value was retained only when the corresponding source evidence supported both the numeric value and its experimental-context assignment. Extracted values with unresolved material, curve/series, panel, dose, time, or endpoint context were not accepted as valid final toxicity records. The complete multimodal extraction workflow is summarized in Fig. 2.

**Fig. 2.**
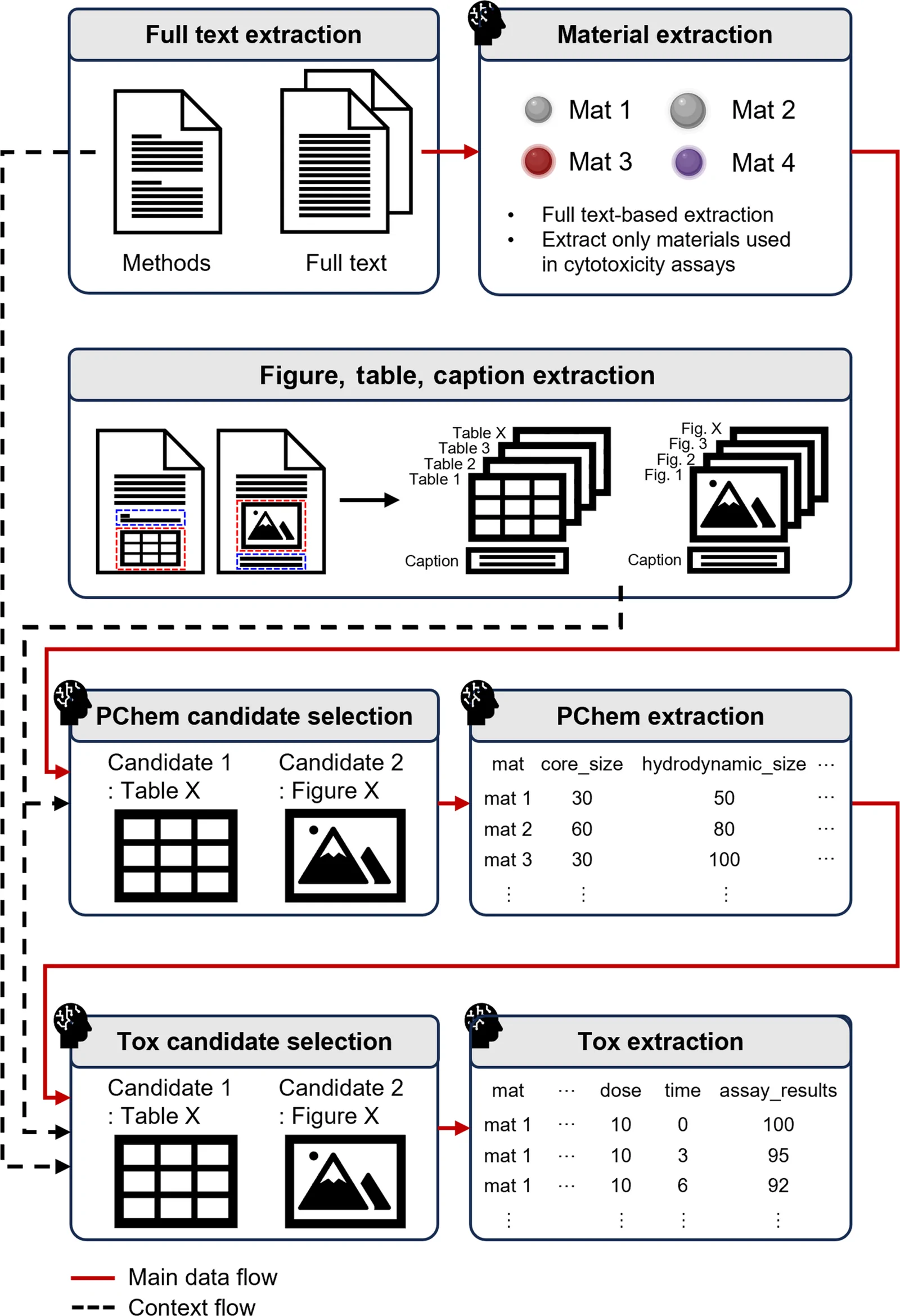
Schematic overview of the data extraction pipeline.

All outputs were saved as structured Excel files, including Mat records, PChem and Tox records, and event logs. Runtime was recorded at both section and batch levels, and logs included detailed metadata (captured stage, status, error type, API call name, payload size, source page, caption length, and number of images) to support traceability and reruns.

### Extraction target and record schema

Extraction targets were organized into 3 groups: Mat, PChem, and Tox attributes. Mat extraction included material identity, composition, and media. PChem extraction included the value, measurement method, and source for 4 key properties—core size, hydrodynamic size, surface charge, and surface area—yielding 12 PChem-related extraction attributes in the final evaluation schema. Tox extraction included assay type, cell name, cell species, cell organ, cell type, exposure time, exposure dose, and assay results.

Not all modeling descriptors were obtained directly from the multimodal extraction workflow. In addition to the extracted physicochemical and toxicological attributes, quantum-mechanical descriptors were incorporated from external literature sources, following the same strategy used in the previous study. Specifically, the enthalpy of formation (Δ*H*_sf_) was obtained from previously reported thermodynamic data for metal oxides [25–32], whereas the conduction band energy (*E*_c_), valence band energy (*E*_v_), and electronegativity of the metal oxide (*χ*_MeO_) were obtained from previously reported band energy and electronegativity data [33–38]. These externally sourced descriptors were included because previous metal oxide nanoSAR studies have linked intrinsic electronic and thermodynamic properties of oxide nanoparticles to toxicity mechanisms and toxicity classification. In particular, conduction-band alignment with cellular redox potentials has been associated with electron-transfer feasibility, reactive oxygen species generation, oxidative stress, and pulmonary inflammation [33], while structure–activity modeling of metal oxide nanoparticles has evaluated energy/enthalpy and electronic descriptors, including atomization energy, Δ*H*_sf_, *E*_c_, *E*_v_, and *χ*_MeO_, as candidate descriptors for toxicity classification [25]. Thus, the present workflow was used to recover literature-derived material, physicochemical, and toxicological records, while selected intrinsic material descriptors were supplemented from previously reported reference datasets for downstream modeling. A representative schema of the output fields, distinguishing automatically extracted Mat/PChem/Tox records from externally supplemented quantum-mechanical descriptors, is shown in Fig. S2.

### Post-extraction processing and construction of modeling datasets

The raw multimodal extraction output was not used directly for predictive modeling. Instead, extracted records underwent structured post-extraction curation to harmonize assay entries and assemble structured datasets for downstream modeling. This curation step included standardization of assay names, retention of direct toxicity-related endpoints with exclusion of mechanistic or otherwise unrelated readouts, filtering based on axis-title information, removal of implausible values, harmonization of cell names, and completion of auxiliary cell information where supported by the extracted context. Assay responses were then normalized to a common 0 to 100 viability-oriented scale. This normalization was performed using source-context-based endpoint interpretation rather than by applying a single numerical rule to all extracted values. Toxicity records were grouped by source context, including PDF key, material, assay type, cell line, source page, source caption, panel label, *x*-axis title, and *y*-axis title. For each source-context group, the scale and semantic meaning of the response were interpreted as fraction versus percentage and viability versus cytotoxicity/cell death. Retained values were then converted to the 0 to 100 viability-oriented scale using deterministic rules: Viability percentages were retained as reported, viability fractions were multiplied by 100, cytotoxicity or cell-death percentages were converted using 100−*x*, and cytotoxicity or cell-death fractions were converted using 100−100*x*. Ambiguous, nondirect, or raw signal-like readouts, such as fold-change or unsupported fluorescence/intensity values, were excluded rather than forced into the viability scale. In addition, zero-dose control rows were inserted for condition groups in which a corresponding control was expected but not recovered during extraction. These rows were deterministically generated only when no zero-dose record was present within the same material, exposure medium, cell line, assay type, and exposure-time group, and only when the endpoint was interpretable on the normalized 0 to 100 viability-oriented scale. The inserted rows were assigned exposure dose = 0 and assay response = 100, corresponding to the untreated reference condition, and were explicitly flagged as synthetic control rows. No synthetic controls were inserted for ambiguous endpoints, raw signal values, or records for which normalization to untreated controls could not be inferred. Following this post-extraction curation, the same dataset-quality procedure used in the previous study was applied to construct the final modeling datasets [13]. This procedure included gap filling and PChem score-based filtering. PChem scores were adopted from the physicochemical data quality scoring framework reported in the previous study [20,39], in which higher scores indicate more complete physicochemical characterization.

### Benchmark design and evaluation of data extraction

Extraction performance was benchmarked on a curated 17-paper oxide nanotoxicity corpus derived from Ha IIIB. For the primary comparative analysis, 15 papers with standard literature layouts were used for aggregate benchmarking, whereas 2 papers containing unusually dense and complex figures were analyzed separately as technical stress-test cases in the Supplementary Materials (Fig. S3).

Performance was evaluated by exact matching for string-valued attributes. For numeric attributes, physicochemical properties, exposure time, and exposure dose were evaluated using a 10% relative error tolerance, with exact matching required for zero-dose records, whereas assay results normalized to the 0 to 100 viability-oriented scale were evaluated using an absolute tolerance of 5%p. To examine whether the resulting performance estimates were sensitive to these threshold choices, we performed a tolerance-sensitivity analysis for the 2 figure-derived toxicological numeric attributes expected to be most affected by tolerance settings: exposure dose and assay results. Exposure dose metrics were recalculated across relative error tolerances of 2%, 4%, 6%, 8%, 10%, and 20%, whereas assay result metrics were recalculated across absolute tolerances of 1%p, 2%p, 3%p, 4%p, 5%p, and 10%p. For each attribute, extracted values were classified as true positives (TP), false positives (FP), false negatives (FN), or true negatives (TN), depending on whether an existing value was correctly extracted, incorrectly extracted, or not extracted, a nonexisting value was incorrectly extracted, or a nonexisting value was correctly returned as “None” (Table 1).

**Table 1.** Overview of classification criteria for evaluating data extraction performance

Actual	Extracted	Classification	Definition
Present	Correctly extracted	TP	A value exists in the ground truth and is correctly extracted.
Present	Incorrectly extracted or not extracted	FN	A value exists in the ground truth, but it is either extracted incorrectly or not extracted.
Absent	Present	FP	No value exists in the ground truth, but a value is extracted.
Absent	“None”	TN	No value exists in the ground truth, and the extraction is correctly returned as “None”.

For toxicological variables, particularly exposure dose and assay results, evaluation was performed at the condition level rather than by isolated numerical matching. Extracted records were sorted by PDF key, material, assay type, cell line, exposure time, and exposure dose, and the correspondence between extracted values (ET) and ground truth values (GT) was manually verified using the original source figure/table context. Matched ground truth values were then entered into newly added Dose (GT) and Results (GT) columns, and TP/FP/FN/TN labels were assigned using formula-based decision rules. Extracted records without a matching ground truth condition were internally encoded as 9999 in the Dose (GT) column and classified as FP; when the dose decision was FP, the corresponding assay result decision was also classified as FP. Synthetic zero-dose control rows were flagged and excluded from extraction-performance metric calculation. An example of this condition-level adjudication procedure is shown in Fig. S4. Precision, recall, accuracy, and F1 score were then calculated from these 4 categories.

### AutoML-based nanotoxicity prediction modeling

Nanotoxicity prediction models were developed using 4 AutoML platforms: Azure [40], Dataiku [41], SageMaker [42], and Vertex AI [43]. For downstream modeling, the extracted datasets were organized into 15 predictor descriptors, whereas assay results were used to define the binary Toxicity endpoint rather than being included as input features. Specifically, assay results of 50 or higher were classified as “Nontoxic”, and values below 50 were classified as “Toxic”. This cutoff was used as a literature-supported operational endpoint for binary nanoSAR classification, not as a claim that smaller viability reductions or assay-response changes above the 50% cutoff are biologically negligible or toxicologically unimportant [13,20,44]. A value below 50% indicates that more than half of the viable cell population was lost relative to the control, which is conceptually aligned with the half-maximal response framework commonly used in toxicology; however, the resulting labels should be interpreted as operational modeling endpoints rather than definitive biological safety classifications.

Before AutoML model training, missing numerical descriptor values were handled using a 2-step gap-filling procedure to generate complete descriptor matrices. For core size and specific surface area, missing values were first estimated from the corresponding available descriptor and material-specific density under a spherical-particle approximation. This step was used because core diameter and specific surface area are physically linked under an idealized spherical geometry. Specific surface area was calculated as $\mathrm{SSA}=6,000/\left(\rho \times d\right)$, where SSA is the specific surface area in m^2^/g, ρ is the density in g/cm^3^, and d is the core diameter in nm. Conversely, missing core size was calculated as $d=6,000/\left(\rho \times \mathrm{SSA}\right)$ when specific surface area was available. In the raw dataset before core size/specific surface area gap filling, surface area was missing in 11,125 of 16,786 records, but core size was available for 10,027 of these surface-area-missing records. In the reciprocal case, 162 records had surface area available, but core size was missing. After spherical-particle gap filling, missingness in both core size and surface area was reduced to 1,098 records. Remaining missing numerical values were imputed using the median of the corresponding descriptor. Median imputation was selected as a conservative and reproducible default rather than as a performance-optimized imputation model. This gap-filling procedure was used to provide complete numerical descriptor matrices for AutoML-based model training. Descriptor completion was performed before train–test partitioning as a dataset-construction step to reduce preprocessing-driven differences among Park-OxideNM2026-derived dataset variants; therefore, the AutoML results should be interpreted as internally controlled comparisons of dataset-construction strategies rather than deployment-level validation based on training-set-locked descriptor completion.

For each dataset, the data were divided into a training set (60%) and a test set (40%). This split ratio was retained for consistency with previous OxideNM nanoSAR and AutoML studies, in which oxide datasets were evaluated using a 60% training set and a 40% test set. The training set was used for model development and cross-validation, whereas the test set was reserved for performance evaluation. Because the Toxic/Nontoxic endpoint was imbalanced, the train–test split was performed using stratified random sampling, with the binary toxicity endpoint used as the stratification variable. To reduce the effect of class imbalance during model development, the Synthetic Minority Over-sampling Technique (SMOTE) was applied only to the training set [45]. The test set was not oversampled and retained its original class distribution. AutoML training settings were specified separately for each platform according to the available platform-specific configuration options. In Azure AutoML, all supported model families were enabled, weighted area under the curve (AUC) was used as the primary optimization metric, validation was handled using the platform auto setting, the user-provided held-out test set was used for final evaluation, and early stopping was enabled. In SageMaker Autopilot, binary classification models were trained using the default F1 objective metric, automatic training mode, an 80:20 internal training-validation split within the training data, and a maximum of 100 model candidates. In Vertex AI, AutoML tabular classification was performed using random data splitting, area under the receiver operating characteristic curve (AUC-ROC) as the optimization target, a 1-h training budget, and early stopping enabled. In Dataiku, 2-class classification was performed using explicit training and test extracts, no additional platform-level sampling, F1 score-based hyperparameter optimization, F1 score-based threshold optimization, grid-search hyperparameter tuning, and 5-fold stratified cross-validation with random seed 1337. Across all platforms, the target column was the binary “toxicity” label derived from assay results. To assess whether synthetic zero-dose control rows affected downstream prediction performance, an ablation analysis was performed using Dataiku as the representative modeling framework. Models were trained and evaluated with and without the flagged synthetic zero-dose control rows using the same train–test split strategy, preprocessing settings, hyperparameter optimization procedure, and evaluation metrics as the main Dataiku workflow. Random Forest, Gradient Boosted Trees, and LightGBM were compared because they showed consistently strong performance across the Park-OxideNM2026-derived datasets in the Dataiku algorithm comparison.

In the comparison of models for individual target materials, the test set for each material was restricted to records corresponding to that material. To maintain independence between training and evaluation data, records assigned to the target-material test set were removed from the corresponding Park-OxideNM2026 and Park-OxideNM2026-Top4 training sets. The multi-material models were then evaluated using the same target-material test records as the model trained only on that material.

### Feature importance and AD analysis

To interpret model behavior, feature importance was quantified using SHAP (Shapley additive explanations) for the best-performing model selected for each dataset on each AutoML platform and summarized as mean percentage contributions across datasets and platforms [46]. For each model, SHAP values were used to estimate the relative contribution of each descriptor to nanotoxicity prediction, and these contributions were then averaged to identify the most influential variables across modeling environments.

AD analysis was performed to compare the descriptor-space coverage of the full OxideNM dataset and its subsets in a common reference space. The AD feature space included both numerical and categorical descriptors. Numerical descriptors included core size, hydrodynamic size, surface charge, surface area, Δ*H*_sf_, *E*_c_, *E*_v_, *χ*_MeO_, exposure time, and exposure dose. Categorical descriptors included material type, cell assay, cell line, cell species, cell origin, and cell type.

The full original dataset, Park-OxideNM2026, was used as the reference dataset. The Top4 and single-material ADs were then reconstructed by filtering this same original full dataset for the corresponding material groups: ZnO, TiO_2_, Fe_3_O_4_, and SiO_2_. This approach ensured that all ADs were compared within the same experimental reference space and that resampling procedures did not artificially expand the estimated AD.

Numerical descriptors with strongly right-skewed non-negative distributions, namely, core size, hydrodynamic size, surface area, exposure time, and exposure dose, were transformed using a $\log\left(1+x\right)$ transformation. All numerical descriptors were then standardized using the mean and standard deviation estimated from the transformed full Park-OxideNM2026 dataset. The same transformation and scaling parameters were applied to all subsets. Categorical descriptors were converted to one-hot encoded variables using category levels observed in the full Park-OxideNM2026 dataset. To reduce the influence of high-cardinality categorical descriptors, such as cell line, each one-hot encoded block was weighted by $1/\sqrt{k}$, where k is the number of categories in that descriptor. The final AD feature matrix was constructed by concatenating the standardized numerical descriptors and the block-weighted categorical descriptors.

Principal components analysis (PCA) was fitted using the full Park-OxideNM2026 feature matrix, and all subset datasets were projected into the same PCA space [47]. AD boundaries were then estimated in the PC1–PC2 space using convex hulls of the projected samples [48]. The convex-hull area was used as the relative AD size and was expressed as a percentage of the hull area of the full Park-OxideNM2026 dataset. The convex-hull area was calculated as:

$$A_{j}=\text{Area}\left(\text{ConvexHull}\left\{\left(\mathrm{PC}1_{i},\;\mathrm{PC}2_{i}\right):i\in D_{j}\right\}\right)$$ (1)

where $D_{j}$ denotes dataset j. To enable direct comparison across datasets, each hull area was normalized relative to the hull area of the full Park-OxideNM2026 dataset:

$$A_{j,\mathrm{rel}}=100\times \frac{A_{j}}{A_{\text{Full}}}$$ (2)

## Results and Discussion

### Evaluation of multimodal nanotoxicity data extraction

Our previous LangChain workflow relied on chunked text retrieval, in which each paper was divided into short text segments and queried using text-only evidence [13]. This strategy was effective for extracting information explicitly stated in the main text, such as material identity and selected physicochemical descriptors, but it was inherently limited for toxicological assay variables, which are often reported in figures and tables, including exposure dose, exposure time, and quantitative assay outcomes. To address this limitation, we redesigned the extraction process as a multimodal, page-preserving workflow. In this revised pipeline, material (Mat) extraction remained largely text-based, whereas physicochemical (PChem) and toxicological (Tox) extraction integrated full-document text, Materials and Methods sections, and figure/table images with their associated captions, rather than relying on early chunk-level retrieval. In the nanotoxicity literature, critical evidence linking NM identity, exposure conditions, and assay responses is often distributed across separate textual and visual components. Preserving that context allowed these previously manual endpoints to be extracted automatically and allowed model performance to be evaluated on quantitative assay conditions and outcomes.

To evaluate the performance of multimodal nanotoxicity data extraction, we compared 4 LLMs (Claude Sonnet 4.6, Gemini 3.1 Pro, GPT-5.4, and Grok 4.20) within the same extraction pipeline. Because all other components were held constant, differences in extraction quality primarily reflect model-specific behavior, while runtime differences reflect overall system performance. The evaluation set consisted of 17 papers, of which 15 were used for the main comparative analysis (Fig. 3B to D). Two papers containing unusually dense and complex figures were analyzed separately from the nominal-layout aggregate metrics, because they were intended to define stress-test boundary conditions; results for the full dataset are provided in Fig. S3.

**Fig. 3.**
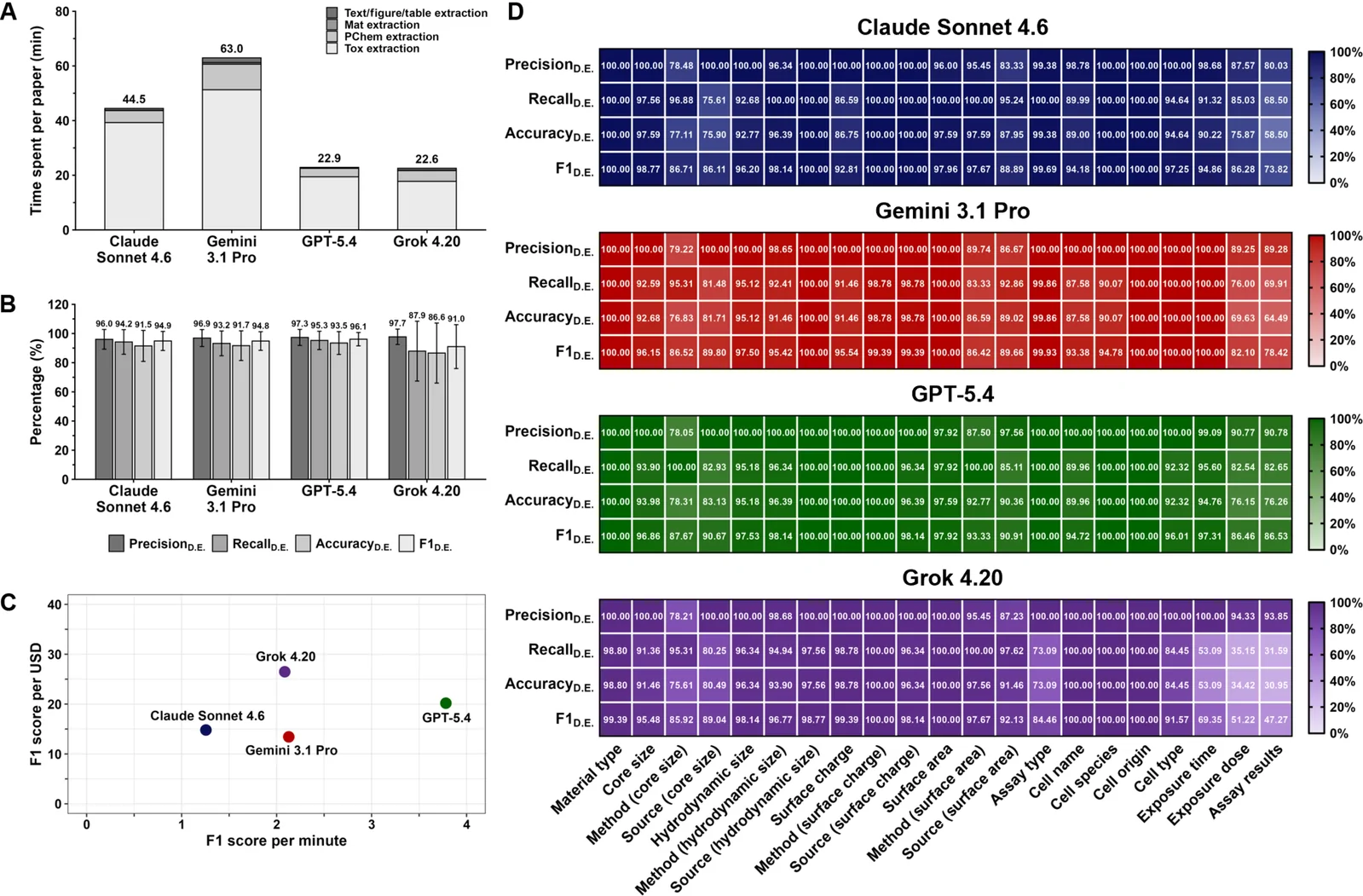
Comparative evaluation of multimodal data extraction performance and practical efficiency. Four LLMs were evaluated and compared in terms of (A) mean extraction time per paper, with stacked bars indicating extraction steps; (B) mean Precision_D.E._, Recall_D.E._, Accuracy_D.E_., and F1_D.E._ across evaluated attributes, with error bars indicating standard deviations; (C) time- and cost-normalized efficiency based on the F1_D.E._ per minute and per USD for the “Assay results” attribute; and (D) performance across individual material, physicochemical, and toxicological attributes.

Model suitability for large-scale data extraction depends not only on extraction quality but also on processing efficiency. We therefore first compared extraction time across models. Mean extraction time per paper differed substantially among models (Fig. 3A). GPT-5.4 and Grok 4.20 were the fastest, requiring 22.9 and 22.6 min per paper, respectively, whereas Claude Sonnet 4.6 and Gemini 3.1 Pro required 44.5 and 63.0 min. Across all models, Tox extraction accounted for the largest share of total runtime, indicating that figure-based data recovery represents the primary computational bottleneck of the pipeline.

Average extraction performance across attributes was high for all 4 models (Fig. 3B), but the mean values masked important endpoint-level differences (Fig. 3D and Fig. S3). GPT-5.4 achieved the highest mean F1_D.E._ (96.1%), while Claude Sonnet 4.6 and Gemini 3.1 Pro showed comparable performance (94.9% and 94.8%, respectively). Grok 4.20 showed the highest mean Precision_D.E._ (97.7%) but lower Recall_D.E._ (87.9%) and Accuracy_D.E._ (86.6%), indicating that high precision alone does not ensure robust extraction performance. At the attribute level, material identity and most PChem descriptors were extracted with near-ceiling performance across models. In contrast, the largest intermodel differences occurred for toxicological endpoints presented in figures, particularly exposure dose and assay results. A tolerance-sensitivity analysis further showed that exposure dose metrics were largely stable across the tested relative error thresholds, whereas assay result metrics increased under more permissive absolute tolerances, as expected; however, GPT-5.4 retained the strongest assay results performance across the tested thresholds in the primary benchmark (Fig. S6). This distinction is important because these endpoints required manual handling in our previous text-based workflow. Assay results extraction showed the clearest separation among models, with F1_D.E._ of 86.53% for GPT-5.4, 78.42% for Gemini 3.1 Pro, 73.82% for Claude Sonnet 4.6, and 47.27% for Grok 4.20. These results suggest that overall mean metrics alone do not fully capture practical performance on the most challenging extraction targets.

When both efficiency and extraction accuracy were considered (Fig. 3C and Table S1), GPT-5.4 provided the most balanced performance. It achieved the highest time-normalized efficiency (3.78 assay results F1 score/min) while maintaining the best assay results extraction performance. Gemini 3.1 Pro showed competitive overall accuracy, but lower efficiency due to longer runtime. Grok 4.20 achieved the highest cost-normalized efficiency (26.49 assay results F1 score/USD), but showed substantially lower assay results performance, which may limit its suitability for reliable dataset construction. Claude Sonnet 4.6 showed intermediate performance in both accuracy and efficiency and did not offer a clear overall advantage over GPT-5.4 within this benchmark. Overall, these results show that multimodal nanotoxicity extraction can achieve high average accuracy, but model choice becomes especially important when figure-based quantitative assay endpoints are included. On this basis, GPT-5.4 was selected as the primary model for downstream large-scale extraction.

### Datasets used for model development

To prepare datasets for downstream nanotoxicity modeling, the multimodally extracted Park-OxideNM2026 records were organized into 4 multi-material datasets with distinct characteristics in terms of coverage, physicochemical data quality, and material composition (Table 2): (a) the full dataset (Park-OxideNM2026), (b) the PChem score-filtered subset (Park-OxideNM2026-HQ), (c) the 4-material subset (Park-OxideNM2026-Top4), and (d) the balanced 4-material subset (Park-OxideNM2026-Top4 B). This dataset design allowed systematic evaluation of how dataset composition—specifically coverage, data quality, material restriction, and class balance—affects downstream predictive performance.

**Table 2.** Construction and characteristics of OxideNM datasets used in the study

Materials	Dataset name	No. rows	No. descriptors	PChem score	Dataset description	Descriptors
All oxide NMs	Park- OxideNM2026	16,786	15	3.2 ± 1.4	- Automatically extracted from text and images in 465 articles. - Gap-filled and used as the full dataset.	Core size, Hydrodynamic size, Surface charge, Surface area, Δ*H*_sf_, *E*_c_, *E*_v_, *χ*_MeO_, Assay type, Cell line, Cell species, Cell organ, Cell type, Exposure time, Exposure dose
	Park- OxideNM2026- HQ	6,609	15	4.7 ± 0.3	- PChem score-filtered subset of Park-OxideNM2026 defined by PChem score ≥ 4. - Gap-filled and used to prioritize physicochemical data quality.	
ZnO, TiO_2_, Fe_3_O_4_, SiO_2_	Park- OxideNM2026- Top4	11,369	15	3.1 ± 1.4	- Subset of Park-OxideNM2026 containing the 4 most represented OxideNMs. - Gap-filled and used to evaluate prediction performance within high-frequency materials.	
	Park- OxideNM2026- Top4 B	7,432	15	3.1 ± 1.4	- Subset of Park-OxideNM2026-Top4 constructed by matching the number of records across 4 OxideNMs. - Gap-filled and used to evaluate the effect of balancing the number of data by material.	

Park-OxideNM2026 served as the broad-coverage reference dataset. It was generated from 465 OxideNM articles and contained 16,786 gap-filled records with 15 predictor descriptors. Among these records, 2,131 rows, corresponding to 12.7% of Park-OxideNM2026, were flagged as synthetic zero-dose controls generated according to the criteria described in the Post-extraction processing and construction of modeling datasets section. Because it retained all extracted OxideNM records, it was used to assess general oxide-wide predictive performance. Its mean PChem score was 3.2 ± 1.4, indicating broad inclusion of records with varying degrees of physicochemical completeness. The material composition and distributions of representative numerical descriptors in Park-OxideNM2026 are shown in Fig. S7.

Quality filtering produced a smaller but more physicochemically complete dataset. Park-OxideNM2026-HQ was prepared by retaining only records with PChem score ≥ 4, resulting in 6,609 records with a higher mean PChem score of 4.7 ± 0.3. This subset was designed to evaluate whether improved data quality can compensate for reduced dataset size and coverage.

Restriction to the most represented materials provided an intermediate modeling scope. Park-OxideNM2026-Top4 contained 11,369 records corresponding to ZnO, TiO_2_, Fe_3_O_4_, and SiO_2_, the 4 most abundant OxideNMs in the dataset. This subset bridged the full multi-material dataset and single-material dataset used later, allowing evaluation of whether focusing on high-frequency materials preserves predictive performance while reducing material diversity.

Park-OxideNM2026-Top4 B was derived from Park-OxideNM2026-Top4 to examine the effect of balanced material representation. This dataset was reduced to 7,432 records by equalizing the number of samples across ZnO, TiO_2_, Fe_3_O_4_, and SiO_2_. Comparison with the unbalanced Top4 dataset enables evaluation of the trade-off between balanced representation and reduced training data size.

All 4 datasets were gap-filled and modeled using the same 15 predictor descriptors, with assay results used only to define the prediction endpoint. Therefore, differences in downstream model performance can be interpreted mainly as consequences of dataset composition, including coverage, physicochemical data quality, material restriction, and material balancing.

### AutoML-based model development

To evaluate how dataset scope and data quality influence nanotoxicity prediction, AutoML-based classification models were developed using 4 datasets derived from Park-OxideNM2026. These datasets enabled comparison of broad material coverage, PChem score-based quality filtering, restriction to frequently represented materials, and balanced materials. To assess robustness across modeling frameworks, each dataset was evaluated using 4 AutoML platforms (Azure, Dataiku, SageMaker, and Vertex AI) and the best-performing model from each platform was compared using Accuracy_N.P._, Precision_N.P._, Recall_N.P._, and F1_N.P._ (Fig. 4). Because nanotoxicity prediction is an imbalanced classification problem, F1_N.P._ was used as the primary performance metric, while Precision_N.P._ and Recall_N.P._ were used to assess prediction balance. The preprocessing methods used in each AutoML platform and the best-performing algorithm selected for each dataset are reported in Table S2. Briefly, Azure selected an ensemble model composed of XGBoost, Random Forest, and LightGBM across the 4 multi-material datasets, whereas Dataiku selected either Gradient Boosted Trees or LightGBM depending on the dataset. SageMaker and Vertex AI returned platform-managed ensemble models. Platform-level preprocessing differed among platforms, with Azure using scaler ensembles and label encoding, Dataiku using StandardScaler and dummy encoding, and SageMaker/Vertex AI not exposing detailed internal preprocessing steps through their managed AutoML pipelines.

**Fig. 4.**
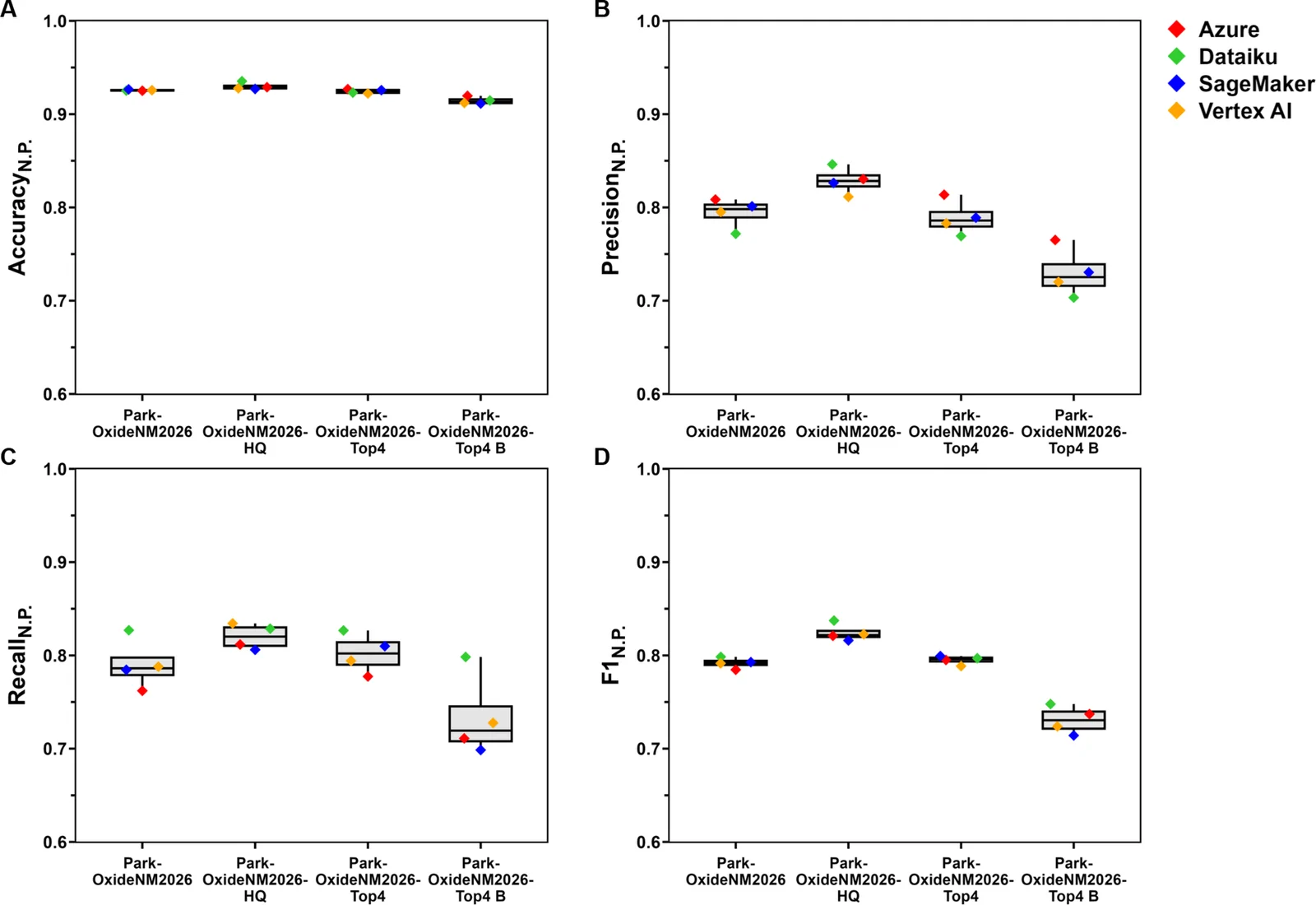
Comparison of AutoML classification performance across 4 Park-OxideNM2026 dataset variants: Park-OxideNM2026, Park-OxideNM2026-HQ, Park-OxideNM2026-Top4, and Park-OxideNM2026-Top4 B. Panels show (A) Accuracy_N.P._, (B) Precision_N.P._, (C) Recall_N.P._, and (D) F1_N.P._. For each dataset, box plots summarize the platform-specific metric values obtained from the best-performing model selected on each AutoML platform, and colored diamonds indicate the individual results for Azure, Dataiku, SageMaker, and Vertex AI.

Across all platforms, Accuracy_N.P._ was consistently high (0.911 to 0.935) but showed limited discriminatory power among datasets (Fig. 4A). In contrast, Precision_N.P._, Recall_N.P._, and F1_N.P._ more clearly reflected the effects of dataset construction (Fig. 4B to D). This pattern is expected for imbalanced nanotoxicity classification problems, in which accuracy alone may overestimate practical model utility [49].

The PChem score-filtered dataset (Park-OxideNM2026-HQ) showed the strongest overall performance. Its mean F1_N.P._ (0.824) exceeded that of the full dataset (0.792), and it also achieved the highest mean Precision_N.P._ (0.829) and Recall_N.P._ (0.820). This improvement was consistent across platforms, with F1_N.P._ of 0.821 (Azure), 0.837 (Dataiku), 0.816 (SageMaker), and 0.823 (Vertex AI). These results indicate that physicochemical data completeness is a key determinant of predictive performance, and that PChem score-based filtering can improve model accuracy despite reducing dataset size [20]. The improvement likely arises from enrichment of records with more reliable and complete descriptors, suggesting that data quality can compensate for reduced data quantity. Consequently, in scenarios where input data resemble those in Park-OxideNM2026-HQ, models trained on PChem score-filtered datasets may be more suitable than those trained on broader, more heterogeneous datasets.

In contrast, material balancing did not improve predictive performance. The balanced dataset (Park-OxideNM2026-Top4 B) showed consistently lower F1_N.P._ than the corresponding unbalanced dataset (Park-OxideNM2026-Top4) across all platforms. Unlike PChem filtering, balancing was achieved through undersampling and was not guided by data quality criteria. As a result, informative samples from overrepresented materials were removed, reducing the effective training data size. These results suggest that the loss of training data outweighed the benefits of improved class balance, indicating that undersampling-based balancing may be detrimental when it disrupts the underlying data distribution. Therefore, Park-OxideNM2026-Top4 B should not be considered a superior alternative to Park-OxideNM2026-Top4 for overall prediction.

Overall, these comparisons indicate that the impact of dataset reduction depends strongly on the selection strategy. PChem score-based filtering improves predictive performance by enhancing descriptor completeness, whereas balancing reduces performance when it removes informative data without improving data quality. Thus, dataset design involves a trade-off between record number, descriptor completeness, and material representation.

Importantly, the same dataset ranking was consistently observed across all AutoML platforms, with Park-OxideNM2026-HQ performing best, Park-OxideNM2026 and Park-OxideNM2026-Top4 showing intermediate performance, and Park-OxideNM2026-Top4 B showing the lowest performance. This consistency indicates that the observed trends are not platform-specific artifacts but reflect intrinsic dataset properties. Among the evaluated platforms, Dataiku achieved the highest or near-highest F1_N.P._ across most datasets and showed consistently strong Recall_N.P._ performance. Figure S8 further summarizes the classification performance obtained for individual algorithms using Dataiku across the 4 multi-material datasets, reporting Accuracy_N.P._, Precision_N.P._, Recall_N.P._, and F1_N.P._ for each combination of algorithm and dataset. Based on this stable and high-performing profile, Dataiku was selected for subsequent material-specific modeling analysis.

Using Dataiku as the representative framework, we evaluated whether the inserted zero-dose control rows biased downstream prediction performance. This ablation analysis compared models trained with and without these controls using Random Forest, Gradient Boosted Trees, and LightGBM (Table S3). The controls slightly increased Accuracy_N.P._ but did not improve F1_N.P_.: F1_N.P._ decreased for Random Forest (0.756 to 0.741) and LightGBM (0.795 to 0.784), and remained nearly unchanged for Gradient Boosted Trees (0.797 to 0.798). Precision_N.P._ also decreased, indicating that the controls did not inflate primary predictive performance but supported restoration of the untreated baseline implicit in normalized dose–response assays.

### Multi- versus single-material model development

Although the previous section evaluated overall performance across multi-material dataset designs, it did not address which modeling strategy is most appropriate when the target material is known. In practical nanotoxicity prediction, the assessment target is often a specific NM with defined physicochemical properties. We therefore compared 3 model scopes—broad multi-material (Park-OxideNM2026), focused multi-material (Park-OxideNM2026-Top4), and single-material models—to determine the optimal strategy for target-specific prediction.

Four single-material datasets were constructed from Park-OxideNM2026 by retaining records corresponding to ZnO, TiO_2_, Fe_3_O_4_, or SiO_2_ (Table 3), the most frequently represented materials in the dataset. For each material, models were evaluated using the same target-specific test set to ensure direct comparability. Figure S9 shows the comparison across evaluated classification algorithms for Park-ZnO, Park-TiO2, Park-Fe3O4, and Park-SiO2.

**Table 3.** Characteristics of single-material datasets used for material-specific nanotoxicity modeling

Materials	Dataset name	No. rows	No. descriptors	Dataset description	Descriptors
ZnO	Park-ZnO	4,708	11	Subset of Park-OxideNM2026 containing only ZnO, the most frequently represented OxideNM.	Core size, Hydrodynamic size, Surface charge, Surface area, Assay type, Cell line, Cell species, Cell organ, Cell type, Exposure time, Exposure dose
TiO_2_	Park-TiO2	2,694	11	Subset of Park-OxideNM2026 containing only TiO_2_, the second most frequently represented OxideNM.	
Fe_3_O_4_	Park-Fe3O4	2,109	11	Subset of Park-OxideNM2026 containing only Fe_3_O_4_, the third most frequently represented OxideNM.	
SiO_2_	Park-SiO2	1,858	11	Subset of Park-OxideNM2026 containing only SiO_2_, the fourth most frequently represented OxideNM.	

Single-material models showed the highest F1_N.P._ across all 4 target materials (Fig. 5D). For ZnO, the improvement was modest (0.79 to 0.81), reflecting its strong representation in the full dataset. In contrast, larger gains were observed for TiO_2_ (0.79 to 0.83), Fe_3_O_4_ (0.72 to 0.79), and SiO_2_ (0.72 to 0.82). These results indicate that restricting training data to the target material improves predictive performance, particularly when the target material is not dominant in the multi-material dataset.

**Fig. 5.**
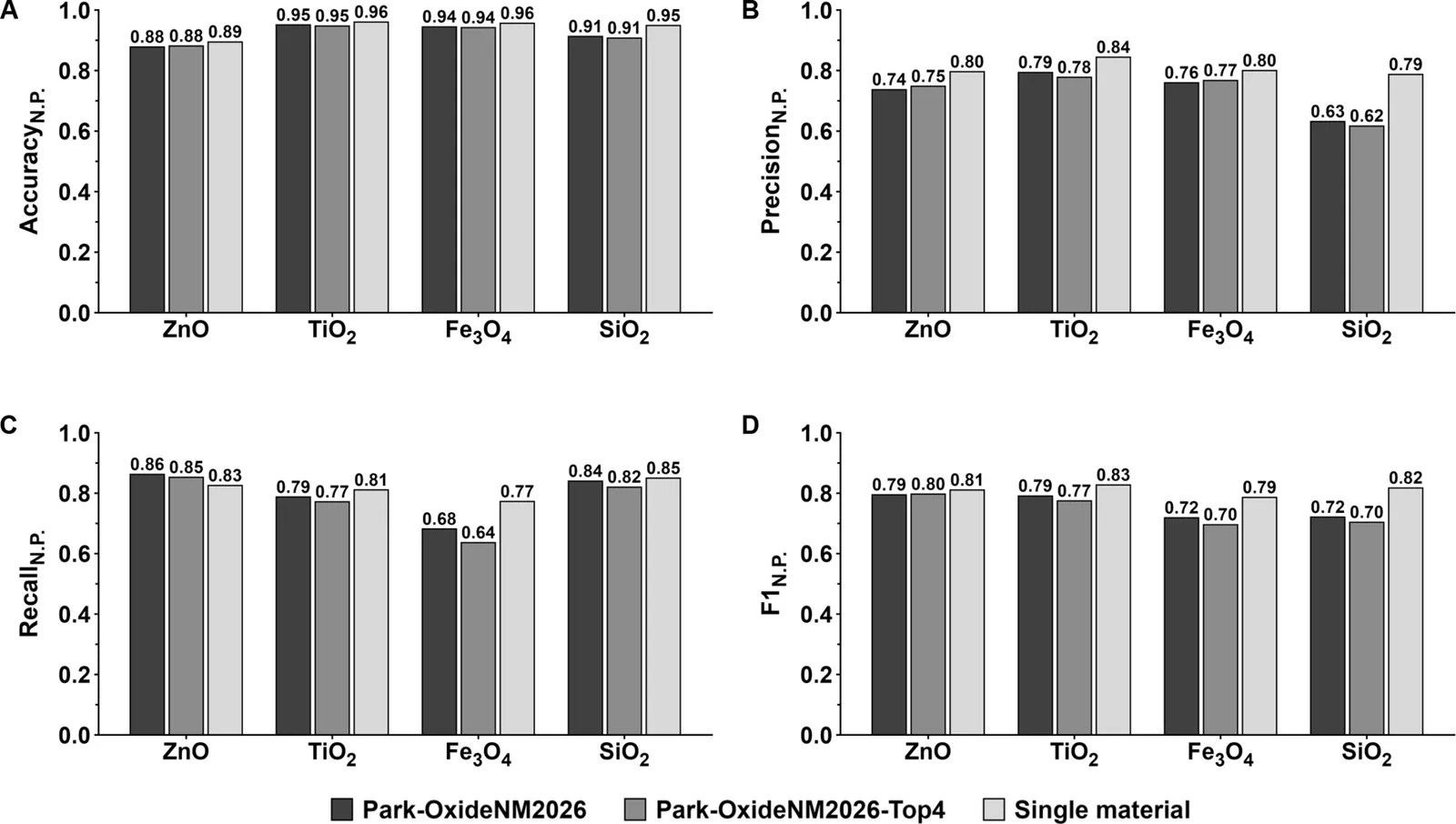
Comparison of nanotoxicity prediction performance across multi-material and single-material models. Models developed using Dataiku were evaluated for ZnO, TiO_2_, Fe_3_O_4_, and SiO_2_ in terms of (A) Accuracy_N.P._, (B) Precision_N.P._, (C) Recall_N.P._, and (D) F1_N.P._. Models were trained on Park-OxideNM2026 (dark gray), Park-OxideNM2026-Top4 (gray), and the corresponding single-material dataset (light gray).

This trend was consistent across multiple evaluation metrics. Single-material models also showed the highest Accuracy_N.P._ and Precision_N.P._ for all 4 materials. Notably, Precision_N.P._ increased substantially for SiO_2_ (0.63 to 0.79), and both Precision_N.P._ and Recall_N.P._ improved for Fe_3_O_4_, indicating that the performance gains were not driven by a single metric.

The improved performance of the single-material models likely reflects reduced heterogeneity in the training data. In the multi-material models, the classifier must learn toxicity patterns across OxideNMs with different intrinsic properties, physicochemical profiles, assay distributions, and dose–response behaviors [6,33,50]. These differences among materials can weaken relationships that are specific to a given target material, particularly when the target material is not dominant in the training set [51]. In contrast, single-material models exclude records from other oxide materials and allow the classifier to focus on variation within the target material, including differences in dose, exposure time, assay type, cell context, and physicochemical conditions. Thus, the higher F1_N.P._ of the single-material models is best interpreted as improved fitting within the target-material data space rather than broader generalization across OxideNMs.

This interpretation is further supported by comparison with Park-OxideNM2026-Top4. Although Park-OxideNM2026-Top4 restricted the training data to the same 4 frequently represented materials, it still combined multiple material identities within a single model. Therefore, the Top4 model reduced the overall material scope but did not eliminate differences among materials. The single-material models provided a more focused learning space by excluding records from other materials entirely, which may explain why they outperformed the Top4 model for all 4 target materials.

The size of the performance gain also differed among materials. ZnO showed only a small difference between the multi-material and single-material models, probably because the large number of ZnO records in Park-OxideNM2026 allowed the multi-material models to learn ZnO-related patterns sufficiently. In contrast, TiO_2_, Fe_3_O_4_, and SiO_2_ showed larger gains from the single-material training. This pattern suggests that models trained only on the target material may be particularly useful when the material is sufficiently represented for separate model development but is less dominant in the broader multi-material training set.

Overall, these results support a data-aware model-selection strategy. The Park-OxideNM2026 model remains useful as a broad-coverage model for oxide-wide prediction, especially when the target material is unknown or insufficiently represented for separate model development. However, for well-represented materials such as ZnO, TiO_2_, Fe_3_O_4_, and SiO_2_, single-material models provided higher target-specific F1_N.P._ than both the full multi-material model and the Top4 multi-material model. A practical prediction platform may therefore benefit from using a broad model for general screening and switching to a single-material model when adequate target-material data are available. Nevertheless, higher target-specific performance alone does not establish broader model utility. The descriptor-space coverage of each model must also be considered, which motivated the subsequent AD analysis.

### Feature importance and AD analysis

To better understand the behavior of the nanotoxicity prediction models and to assess their robustness in descriptor space, feature importance and the AD analysis were performed across all datasets (Fig. 6). Feature importance was summarized across the AutoML platforms, while ADs were estimated in a common descriptor feature space and visualized by PCA.

**Fig. 6.**
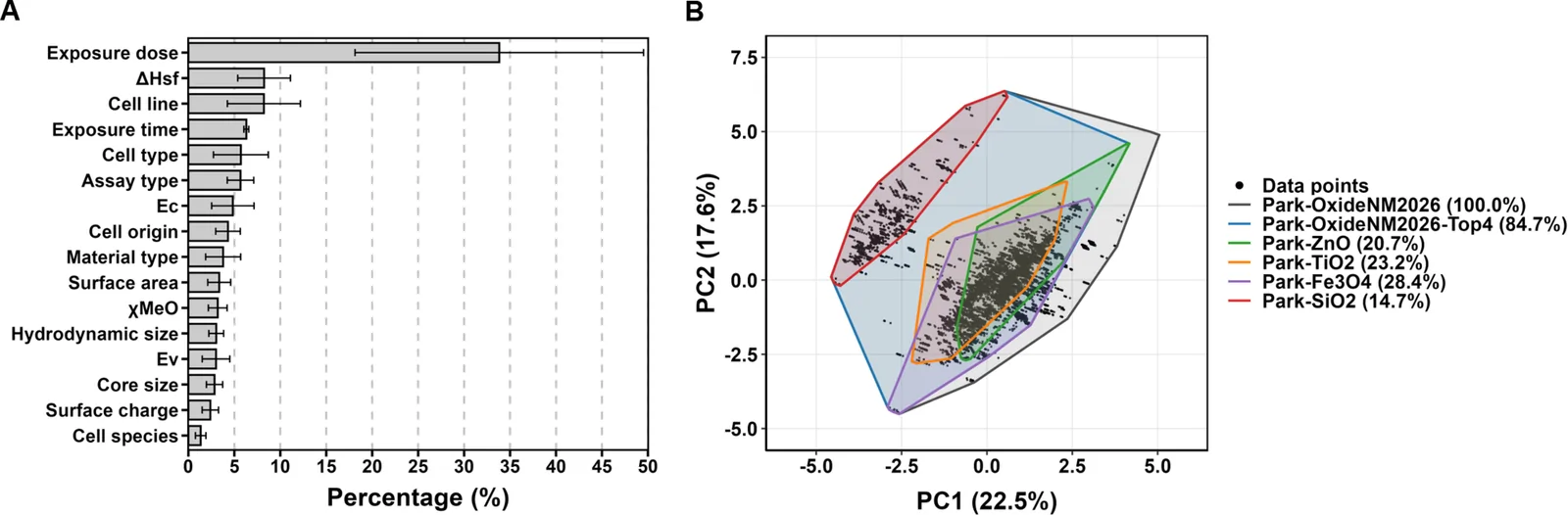
Feature importance and applicability domain of nanotoxicity prediction models. (A) Mean feature importance of the descriptors used in the nanotoxicity prediction models. Bars show the mean percentage contribution of each descriptor across AutoML platforms, and error bars represent standard deviations. (B) PCA plot of the applicability domain feature space for Park-OxideNM2026 and its derived subsets. Black points indicate individual Park-OxideNM2026 records, and colored boundaries show the estimated applicability domains of Park-OxideNM2026 (black), Park-OxideNM2026-Top4 (blue), Park-ZnO (green), Park-TiO2 (orange), Park-Fe3O4 (purple), and Park-SiO2 (red). Percentages in parentheses represent relative domain coverage compared with Park-OxideNM2026.

As shown in Fig. 6A, exposure dose was the most influential descriptor, accounting for the largest mean feature contribution among all variables. This result is consistent with the structure of in vitro cytotoxicity data, where toxicity classification is strongly dependent on the applied dose. Similar machine-learning studies in NM toxicity have also identified exposure dose or concentration, exposure duration, assay type, and cell-related variables as important predictors of in vitro toxicity outcomes, supporting the present observation that experimental attributes can dominate the model predictions [13,52,53]. Other descriptors, including Δ*H*_sf_, cell line, exposure time, cell type, assay type, and *E*_c_, showed smaller but still notable contributions. In contrast, individual physicochemical descriptors such as core size, hydrodynamic size, surface charge, and surface area had lower contributions. These results suggest that the models relied most strongly on exposure conditions, while biological assay context and selected material-intrinsic descriptors also contributed to toxicity classification.

The AD analysis showed that dataset scope strongly affected descriptor-space coverage (Fig. 6B). The full dataset, Park-OxideNM2026, defined the broadest domain (100% relative coverage), while Park-OxideNM2026-Top4 retained 84.7% of this space. This indicates that the 4 most frequently represented materials capture a large portion of the central descriptor space, despite the presence of additional materials in the full dataset. The categorical components contributing to this descriptor-space comparison are summarized in Table S4.

In contrast, the single-material datasets showed substantially narrower ADs with relative coverage of 20.7% (ZnO), 23.2% (TiO_2_), 28.4% (Fe_3_O_4_), and 14.7% (SiO_2_). These results demonstrate that, even with sufficient sample size, single-material datasets inherently cover a limited region of the descriptor space, reflecting reduced diversity in experimental conditions and feature combinations.

This pattern contrasts with the material-wise prediction results in Fig. 5. In that analysis, single-material models showed higher F1_N.P._ than the multi-material models for the corresponding target materials, particularly for Fe_3_O_4_ and SiO_2_. However, the AD analysis shows that these improved target-specific performances were accompanied by narrower descriptor-space coverage. This indicates a practical trade-off between model specificity and applicability. Single-material models can provide improved prediction within a defined target-material domain, but their use is expected to be more restricted with respect to assay conditions and descriptor combinations. Conversely, the full Park-OxideNM2026 model may provide lower target-specific performance for some materials, but it offers broader coverage across the OxideNM descriptor space.

Overall, these results support a data-aware model-selection strategy. The full Park-OxideNM2026 model provides the broadest descriptor-space coverage and is therefore most appropriate when broad oxide-wide screening is needed. In contrast, single-material models provide improved target-specific prediction but operate within narrower material-specific domains. The choice between broad multi-material and single-material models should depend on the intended use. Broad coverage is preferable when the target material or experimental context is uncertain, whereas single-material models may be advantageous when the target material is known and sufficient material-specific data are available.

### Operational scope and future perspectives of multimodal LLMs

First, the operational scope of this framework is structured as a robust 2-stage architecture: a high-density validation phase using a curated 17-paper ground truth corpus, followed by a large-scale production deployment that generated the final Park-OxideNM2026 dataset, comprising 16,786 structured records compiled from 465 verified full-text articles. Within the initial validation phase, the 15-paper primary baseline demonstrated excellent data recovery fidelity on standard literature layouts. To systematically map the performance boundaries of current vision-language models, 2 hyper-complex edge cases—featuring a 17-series overlapping line plot and a dense 3-dimensional bar plot—were deliberately analyzed separately as technical stress tests rather than standard nominal evaluations. These extreme visual encodings revealed that heavy spatial pixel overlap primarily constrains model recall rather than extraction reasoning. This systematic boundary mapping establishes a clear engineering roadmap for future model fine-tuning specifically optimized to parse nonstandard formats, such as heatmaps and quantitative data scattered within unstructured supplementary documents.

Second, while the production-level dataset expansion was intentionally restricted to OxideNMs and in vitro cytotoxicity endpoints to leverage the most data-rich sector of existing literature, the core pipeline architecture is inherently material- and assay-agnostic. The fundamental workflow blocks—including document layout parsing, multimodal prompt compilation, and structured schema enforcement—are fully transferable to more diverse and intricate chemical spaces. Scaling this extraction framework to ingest metallic- and carbon-based NMs, doped systems, or complex in vivo biological profiles requires only updating the targeted database schemas and prompt templates. At the same time, cross-resource external evaluation should be implemented as a schema-harmonized validation step rather than as a simple direct merge with existing databases, because independently curated nanotoxicity resources often use different endpoint definitions, descriptor ontologies, assay-response normalization rules, and source-article coverage. Future deployment should therefore incorporate multi-resource semantic cross-validation after descriptor harmonization and source-literature overlap screening so that extraction fidelity and prediction transferability can be evaluated in genuinely independent database contexts. Overall, this high expandability eliminates the need to redesign or rebuild the underlying document-processing engine, providing a practical and scalable pathway for expanding global, automated nanoinformatics curation networks.

## Conclusion

In this study, we developed a multimodal LLM workflow for automated extraction of oxide nanotoxicity data from scientific literature. By integrating full article text with figures, tables, captions, legends, and Materials and Methods sections, the workflow substantially reduced the manual recovery previously required for quantitative physicochemical and toxicological variables, including exposure conditions and assay responses. Benchmarking of 4 multimodal LLMs identified GPT-5.4 as the most practical model within this workflow, providing the best balance among extraction performance, runtime, and cost. Application of the selected workflow to the 821-paper oxide literature pool, followed by post-extraction screening, yielded 465 in vitro oxide nanotoxicity articles. The corresponding extracted records were compiled into Park-OxideNM2026, a structured and gap-filled dataset containing 16,786 records prepared for modeling.

Park-OxideNM2026 enabled systematic evaluation of how dataset scope and construction strategy affect downstream nanotoxicity prediction. The PChem score-filtered subset, Park-OxideNM2026-HQ, achieved the strongest overall predictive performance despite its smaller size, suggesting that improved physicochemical data completeness can compensate for reduced record number and coverage. In contrast, the material-balanced subset, Park-OxideNM2026-Top4 B, did not improve performance relative to Park-OxideNM2026-Top4, indicating that undersampling to equalize material representation can reduce predictive performance when the loss of training data is not offset by improved data quality. These findings show that dataset reduction is not inherently beneficial or detrimental; its effect depends on whether the subset construction improves descriptor completeness, material representation, or both.

The comparison of model scopes further showed that the appropriate model depends on the intended prediction task. For known and sufficiently represented target materials, including ZnO, TiO_2_, Fe_3_O_4_, and SiO_2_, single-material models provided higher target-specific F1_N.P._ than both the broad Park-OxideNM2026 model and the focused Park-OxideNM2026-Top4 model. However, AD analysis showed that these performance gains were accompanied by substantially narrower descriptor-space coverage. Therefore, the improved performance of single-material models should be interpreted as better fitting within defined target-material domains rather than broader generalization across OxideNMs. Feature importance analysis showed that exposure dose and other experimental-context descriptors played major roles in nanotoxicity prediction, emphasizing the importance of accurately extracting assay conditions as well as material properties.

In conclusion, this study demonstrates that multimodal extraction can support scalable construction of structured nanotoxicity datasets for downstream modeling and enable data-aware model selection. The full Park-OxideNM2026 dataset provides broad descriptor-space coverage for general screening across OxideNMs, whereas PChem score-filtered or single-material datasets may be advantageous when the target prediction domain is well defined and sufficiently represented. Future work should further validate large-scale extracted records, refine post-extraction curation, extend the workflow to additional NM classes and biological endpoints, and develop user-oriented prediction platforms that report both predictive performance and AD coverage.

## Data Availability

The Park-OxideNM2026 dataset produced in this study is available on Zenodo under the identifier https://doi.org/10.5281/zenodo.19910917. The multimodal data extraction pipeline is available at the public GitHub repository: https://github.com/yoon-lab/Nanotoxicity-Data-Extraction-Pipeline2026. The repository includes comprehensive documentation, installation instructions, and sample scripts to ensure full reproducibility of the reported results. Additionally, a permanent archive of the specific code version used in this study has been deposited in Zenodo (DOI: 10.5281/zenodo.20753451) to ensure long-term accessibility. All code is licensed under Apache 2.0, allowing for transparent use and further community-driven development. Legal use depends on the user’s access rights and the applicable publisher license terms.
