# Supplementary material for: Automated Large-Scale Extraction of Nanotoxicity Data from the Literature Using Multimodal Large Language Models
Source: Comput Struct Biotechnol J. 2026 Jul 7;35(3):0161. doi: 10.34133/csbj.0161 (PMC13632934; doi:10.34133/csbj.0161)
Supplement: Supplementary file 1 [file csbj.0161.f1.docx]

**Automated Large-Scale Extraction of Nanotoxicity Data from the Literature Using Multimodal Large Language Models**

**Seung-Geun Park^1^, Seung Min Ha^1^, Zayakhuu Gerelkhuu^2^ and Tae Hyun Yoon^1,2,3,4,*^**

^1^Department of Chemistry, Hanyang University, Seoul 04763, Republic of Korea

^2^Research Institute for Convergence of Basic Science, Hanyang University, Seoul 04763, Republic of Korea

^3^Institute of Next Generation Material Design, Hanyang University, Seoul 04763, Republic of Korea

^4^Yoon Idea Lab. Co. Ltd., Seoul 04763, Republic of Korea

**Correspondence**

Tae Hyun Yoon, Department of Chemistry, Hanyang University, Seoul 04763, Republic of Korea

E-mail: taeyoon@hanyang.ac.kr

**Supporting information**

**Fig. S1.** Prompt and script refinement workflow for multimodal data extraction. The extraction code was iteratively drafted, executed, inspected, queried to the AI assistant, revised, and rerun until the intended structured outputs were obtained. Representative revisions for PChem and Tox extraction are shown.

**Fig. S2.** Representative output schema of the curated dataset. Automatically extracted Mat, PChem, and Tox records include material identity, media, physicochemical properties, assay conditions, response values, methods, units, source labels, and provenance, while externally supplemented quantum-mechanical descriptors are shown separately as manually curated fields.

**Fig. S3.** Heatmaps of data extraction performance across individual material, physicochemical, and toxicological attributes using the full set of 17 source articles.

**Fig. S4.** Example of condition-level source-context adjudication for toxicological extraction evaluation. Extracted toxicity records (ET) were sorted by PDF key, material, assay type, cell line, exposure time, and exposure dose, and then manually matched to ground truth records (GT) using the original source figure/table context. Matched GT values were entered into newly added Dose (GT) and Results (GT) columns, and TP/FP/FN/TN labels were assigned using formula-based decision rules. Dose values were classified as TP when the relative error was ≤10%, with exact agreement required for zero-dose records, while assay results were classified as TP when the absolute difference from GT was ≤5 percentage points on the 0–100 viability scale. Records without a matching GT condition were internally encoded as 9999 and classified as FP; synthetic zero-dose controls were flagged and excluded from extraction-performance metric calculation.

**Fig. S5.** Extraction outcomes for evaluated attributes across four multimodal LLMs. Stacked horizontal bars show the percentage of TP, FP, FN, and TN for each evaluated attribute. Results are shown separately for (A) Claude Sonnet 4.6, (B) Gemini 3.1 Pro, (C) GPT-5.4, and (D) Grok 4.20.

**Fig. S6.** Sensitivity analysis of numeric extraction performance metrics across tolerance thresholds. Precision_D.E._ (blue), Recall_D.E._ (orange), Accuracy_D.E._ (green), and F1_D.E._ (red) were recalculated for (A) exposure dose using relative error tolerances of 2–20% and for (B) assay results using absolute tolerances of 1–10%p. Solid lines indicate the primary benchmark excluding two unusually complex articles, and dashed lines indicate the full benchmark including these articles. Results are shown for Claude Sonnet 4.6, Gemini 3.1 Pro, GPT-5.4, and Grok 4.20.

**Fig. S7.** Material composition and distributions of selected numerical attributes in the dataset. (A) Pie chart showing the relative proportions of material identities represented in the dataset. The most frequent oxide nanomaterials are labeled with percentages. (B) Histograms showing the count distributions of selected numerical attributes, including core size, hydrodynamic size, surface charge, surface area, exposure time, and exposure dose. Units are given in the panel titles, and the y-axis shows the number of entries. For each attribute, the histograms were generated after excluding values above the 95th percentile to reduce the influence of extreme high-end values and improve visualization of the main data distribution. Units are given in the panel titles, and the y-axis shows the number of entries.

**Fig. S8.** Comparison of classification performance for models trained on Park-OxideNM2026-derived multi-material datasets. Heatmaps present (A) Accuracy_N.P._, (B) Precision_N.P._, (C) Recall_N.P._, and (D) F1_N.P._ for models developed with each evaluated classification algorithm in Dataiku. Rows list the algorithms, and columns indicate the modeling datasets, Park-OxideNM2026, Park-OxideNM2026-HQ, Park-OxideNM2026-Top4, and Park-OxideNM2026-Top4 B. Numbers within the cells show the metric value for each algorithm–dataset pair, with darker red indicating higher performance.

**Fig. S9.** Comparison of classification performance for models trained on Park-OxideNM2026-derived single-material datasets. Heatmaps present (A) Accuracy_N.P._, (B) Precision_N.P._, (C) Recall_N.P._, and (D) F1_N.P._ for models developed with each evaluated classification algorithm in Dataiku. Rows list the algorithms, and columns indicate the modeling datasets, Park-ZnO, Park-TiO2, Park-Fe3O4, and Park-SiO2. Numbers within the cells show the metric value for each algorithm–dataset pair, with darker red indicating higher performance.

**Table S1.** Runtime and cost comparison of multimodal LLMs.

**Table S2.** Preprocessing methods and best-performing algorithms used in different AutoML platforms.

**Table S3.** Ablation analysis assessing the effect of synthetic zero-dose control rows on nanotoxicity prediction performance.

**Table S4.** Comparison of the categorical applicability domain across the Park-OxideNM2026, Park-OxideNM2026-HQ, and Park-OxideNM2026-Top4 datasets.

**Figures**

**Fig. S1.** Prompt and script refinement workflow for multimodal data extraction. The extraction code was iteratively drafted, executed, inspected, queried to the AI assistant, revised, and rerun until the intended structured outputs were obtained. Representative revisions for PChem and Tox extraction are shown.

**Fig. S2.** Representative output schema of the curated dataset. Automatically extracted Mat, PChem, and Tox records include material identity, media, physicochemical properties, assay conditions, response values, methods, units, source labels, and provenance, while externally supplemented quantum-mechanical descriptors are shown separately as manually curated fields.

**Fig. S3.** Heatmaps of data extraction performance across individual material, physicochemical, and toxicological attributes using the full set of 17 source articles.

**Fig. S4.** Example of condition-level source-context adjudication for toxicological extraction evaluation. Extracted toxicity records (ET) were sorted by PDF key, material, assay type, cell line, exposure time, and exposure dose, and then manually matched to ground truth records (GT) using the original source figure/table context. Matched GT values were entered into newly added Dose (GT) and Results (GT) columns, and TP/FP/FN/TN labels were assigned using formula-based decision rules. Dose values were classified as TP when the relative error was ≤10%, with exact agreement required for zero-dose records, while assay results were classified as TP when the absolute difference from GT was ≤5 percentage points on the 0–100 viability scale. Records without a matching GT condition were internally encoded as 9999 and classified as FP; synthetic zero-dose controls were flagged and excluded from extraction-performance metric calculation.

**Fig. S5.** Extraction outcomes for evaluated attributes across four multimodal LLMs. Stacked horizontal bars show the percentage of TP, FP, FN, and TN for each evaluated attribute. Results are shown separately for (A) Claude Sonnet 4.6, (B) Gemini 3.1 Pro, (C) GPT-5.4, and (D) Grok 4.20.

**Fig. S6.** Sensitivity analysis of numeric extraction performance metrics across tolerance thresholds. Precision_D.E._ (blue), Recall_D.E._ (orange), Accuracy_D.E._ (green), and F1_D.E._ (red) were recalculated for exposure dose using relative-error tolerances of 2–20% and for assay results using absolute tolerances of 1–10%p. Solid lines indicate the primary benchmark excluding two unusually complex articles, and dashed lines indicate the full benchmark including these articles. Results are shown for Claude Sonnet 4.6, Gemini 3.1 Pro, GPT-5.4, and Grok 4.20.

**Fig. S7.** Material composition and distributions of selected numerical attributes in the dataset. (A) Pie chart showing the relative proportions of material identities represented in the dataset. The most frequent oxide nanomaterials are labeled with percentages. (B) Histograms showing the count distributions of selected numerical attributes, including core size, hydrodynamic size, surface charge, surface area, exposure time, and exposure dose. Units are given in the panel titles, and the y-axis shows the number of entries. For each attribute, the histograms were generated after excluding values above the 95th percentile to reduce the influence of extreme high-end values and improve visualization of the main data distribution. Units are given in the panel titles, and the y-axis shows the number of entries.

**Fig. S8.** Comparison of classification performance for models trained on Park-OxideNM2026-derived multi-material datasets. Heatmaps present (A) Accuracy_N.P._, (B) Precision_N.P._, (C) Recall_N.P._, and (D) F1_N.P._ for models developed with each evaluated classification algorithm in Dataiku. Rows list the algorithms, and columns indicate the modeling datasets, Park-OxideNM2026, Park-OxideNM2026-HQ, Park-OxideNM2026-Top4, and Park-OxideNM2026-Top4 B. Numbers within the cells show the metric value for each algorithm–dataset pair, with darker red indicating higher performance.

**Fig. S9.** Comparison of classification performance for models trained on Park-OxideNM2026-derived single-material datasets. Heatmaps present (A) Accuracy_N.P._, (B) Precision_N.P._, (C) Recall_N.P._, and (D) F1_N.P._ for models developed with each evaluated classification algorithm in Dataiku. Rows list the algorithms, and columns indicate the modeling datasets, Park-ZnO, Park-TiO2, Park-Fe3O4, and Park-SiO2. Numbers within the cells show the metric value for each algorithm–dataset pair, with darker red indicating higher performance.

**Tables**

**Table S1**
Runtime and cost comparison of multimodal LLMs.

|  | Claude Sonnet 4.6 | Gemini 3.1 Pro | GPT-5.4 | Grok 4.20 |
| --- | --- | --- | --- | --- |
| Elapsed time per paper | 44 min 27 sec | 62 min 57 sec | 22 min 53 sec | 22 min 33 sec |
| Spent cost per paper | $5.497 | $5.298 | $4.287 | $1.785 |

**Table S2**
Preprocessing methods and best-performing algorithms used in different AutoML platforms.

|  | Datasets | Azure | Dataiku | SageMaker | Vertex AI |
| --- | --- | --- | --- | --- | --- |
| Preprocessing | Park- OxideNM2026 | Ensemble (StandardScaler, MaxAbsScaler); Label encoding | StandardScaler; Dummy encoding | Not visible | Not visible |
|  | Park- OxideNM2026- HQ | Ensemble (StandardScaler, SparseNormalizer MaxAbsScaler); Label encoding | StandardScaler; Dummy encoding | Not visible | Not visible |
|  | Park- OxideNM2026- Top4 | Ensemble (StandardScaler, MaxAbsScaler); Label encoding | StandardScaler; Dummy encoding | Not visible | Not visible |
|  | Park- OxideNM2026- Top4 B | Ensemble (StandardScaler, SparseNormalizer MaxAbsScaler); Label encoding | StandardScaler; Dummy encoding | Not visible | Not visible |
|  | Park- OxideNM2026- ZnO | - | StandardScaler; Dummy encoding | - | - |
|  | Park- OxideNM2026- TiO2 | - | StandardScaler; Dummy encoding | - | - |
|  | Park- OxideNM2026- Fe3O4 | - | StandardScaler; Dummy encoding | - | - |
|  | Park- OxideNM2026- SiO2 | - | StandardScaler; Dummy encoding | - | - |
| Best performing algorithm | Park- OxideNM2026 | Ensemble (XGBoost, Random Forest, LightGBM) | Gradient Boosted Trees | Ensemble^a^ | Ensemble^b^ |
|  | Park- OxideNM2026- HQ | Ensemble (XGBoost, Random Forest, LightGBM) | LightGBM | Ensemble^a^ | Ensemble^b^ |
|  | Park- OxideNM2026- Top4 | Ensemble (XGBoost, Random Forest, LightGBM) | Gradient Boosted Trees | Ensemble^a^ | Ensemble^b^ |
|  | Park- OxideNM2026- Top4 B | Ensemble (XGBoost, Random Forest, LightGBM) | Gradient Boosted Trees | Ensemble^a^ | Ensemble^b^ |
|  | Park- OxideNM2026- ZnO | - | Gradient Boosted Trees | - | - |
|  | Park- OxideNM2026- TiO2 | - | LightGBM | - | - |
|  | Park- OxideNM2026- Fe3O4 | - | LightGBM | - | - |
|  | Park- OxideNM2026- SiO2 | - | Gradient Boosted Trees | - | - |

^a^SageMaker supports only ensemble models composed of the following algorithms: LightGBM, CatBoost, XGBoost, Random Forest, Extra Trees, linear models, Neural Network Torch, and Neural Network Fast.ai.
^b^Vertex AI for tabular classification supports only ensemble models composed of boosted-tree and neural-network.

**Table S3**
Ablation analysis assessing the effect of synthetic zero-dose control rows on nanotoxicity prediction performance.

| Algorithms | Accuracy | | |  | | Precision | | |  | | Recall | | |  | | F1 | | |
| --- | --- | --- | --- | --- | --- | --- | --- | --- | --- | --- | --- | --- | --- | --- | --- | --- | --- | --- |
|  | With synthetic control | Without synthetic control |  | | With synthetic control | | Without synthetic control |  | | With synthetic control | | Without synthetic control |  | | With synthetic control | | Without synthetic control |  |
| Random Forest | 0.903 | 0.897 |  | | 0.708 | | 0.732 |  | | 0.776 | | 0.783 |  | | 0.741 | | 0.756 |  |
| Gradient Boosted Trees | 0.925 | 0.918 |  | | 0.772 | | 0.815 |  | | 0.827 | | 0.780 |  | | 0.798 | | 0.797 |  |
| LightGBM | 0.921 | 0.915 |  | | 0.766 | | 0.786 |  | | 0.804 | | 0.804 |  | | 0.784 | | 0.795 |  |

**Table S4**
Comparison of the categorical applicability domain across the Park-OxideNM2026, Park-OxideNM2026-HQ, and Park-OxideNM2026-Top4 datasets.

| Attributes | Datasets | Components |
| --- | --- | --- |
| Material type | Park-OxideNM2026 (39) | Ag_2_O, Al_2_O_3_, BaTiO_3_, Bi_2_O_3_, CaO, CeO_2_, Co_3_O_4_, CoFe_2_O_4_, CoO, Cr_2_O_3_, Cu_2_O, CuO, Fe_2_O_3_, Fe_3_O_4_, FeO, Gd_2_O_3_, HfO_2_, In_2_O_3_, La_2_O_3_, MgO, Mn_2_O_3_, Mn_3_O_4_, MnO_2_, MoO_3_, Nd_2_O_3_, Ni_2_O_3_, NiO, Sb_2_O_3_, SiO_2_, SnO_2_, TiO_2_, V_2_O_3_, V_2_O_5_, WO_3_, Y_2_O_3_, Yb_2_O_3_, ZnFe_2_O_4_, ZnO, ZrO_2_ |
|  | Park-OxideNM2026-HQ (26) | Ag_2_O, Al_2_O_3_, BaTiO_3_, CeO_2_, Co_3_O_4_, CoFe_2_O_4_, CoO, Cu_2_O, CuO, Fe_2_O_3_, Fe_3_O_4_, Gd_2_O_3_, HfO_2_, MgO, Mn_2_O_3_, Mn_3_O_4_, NiO, SiO_2_, SnO_2_, TiO_2_, V_2_O_5_, WO_3_, Y_2_O_3_, ZnFe_2_O_4_, ZnO, ZrO_2_ |
|  | Park-OxideNM2026-Top4 (4) | Fe_3_O_4_, SiO_2_, TiO_2_, ZnO |
| Assay type | Park-OxideNM2026 (47) | AK release, Alamar Blue, Annexin V staining, Annexin V/PI, AO/EtBr, ATP, BrdU, Calcein-AM, Calcein/PI, Caspase-3/7 activity, CBMN, CBMN Cytome, CBPI, CC50, CCK-8, CellTiter-Blue, CFE, Coomassie Blue, Crystal Violet staining, CyQUANT, Cytell Cell Viability BioApp, Cytotox Red, CytoTox-Glo, DAPI staining, DRAQ7, FDA, Hemocytometer, Hemolysis assay, Hoechst staining, Hoechst/PI staining, Live/Dead staining, MTS, MTT, NRU, PI staining, PicoGreen, PrestoBlue, Resazurin, RTCA, SRB, SYTOX Green, TOTO-3 exclusion, ToxTracker, Trypan Blue, TUNEL, WST, XTT |
|  | Park-OxideNM2026-HQ (31) | Alamar Blue, Annexin V staining, Annexin V/PI, ATP, Calcein-AM, Calcein/PI, CBPI, CCK-8, CFE, Coomassie Blue, Cytell Cell Viability BioApp, Cytotox Red, DAPI staining, DRAQ7, FDA, Hemocytometer, Hemolysis assay, Hoechst staining, Live/Dead staining, MTS, MTT, NRU, PI staining, PrestoBlue, Resazurin, RTCA, SRB, SYTOX Green, Trypan Blue, WST, XTT |
|  | Park-OxideNM2026-Top4 (45) | AK release, Alamar Blue, Annexin V staining, Annexin V/PI, AO/EtBr, ATP, BrdU, Calcein-AM, Calcein/PI, Caspase-3/7 activity, CBMN, CBMN Cytome, CBPI, CC50, CCK-8, CellTiter-Blue, CFE, Coomassie Blue, Crystal Violet staining, CyQUANT, Cytell Cell Viability BioApp, CytoTox-Glo, DAPI staining, DRAQ7, FDA, Hemocytometer, Hemolysis assay, Hoechst staining, Hoechst/PI staining, Live/Dead staining, MTS, MTT, NRU, PI staining, PicoGreen, PrestoBlue, Resazurin, RTCA, SRB, SYTOX Green, TOTO-3 exclusion, Trypan Blue, TUNEL, WST, XTT |
| Cell organ | Park-OxideNM2026 (34) | Adipose tissue, Adrenal gland, Bladder, Blood, Blood vessel, Bone, Bone marrow, Brain, Breast, Cervix, Colon, Embryo, Esophagus, Eye, Fibroblast, Heart, Kidney, Liver, Lung, Mesothelium, Muscle, Nose, Oral, Other, Ovary, Pancreas, Placenta, Prostate, Skin, Small intestine, Spleen, Stomach, Testis, Thyroid |
|  | Park-OxideNM2026-HQ (30) | Adipose tissue, Adrenal gland, Bladder, Blood, Blood vessel, Bone marrow, Brain, Breast, Cervix, Colon, Embryo, Esophagus, Eye, Fibroblast, Heart, Kidney, Liver, Lung, Mesothelium, Muscle, Nose, Oral, Other, Ovary, Pancreas, Prostate, Skin, Small intestine, Stomach, Testis |
|  | Park-OxideNM2026-Top4 (31) | Adipose tissue, Adrenal gland, Bladder, Blood, Blood vessel, Bone, Bone marrow, Brain, Breast, Cervix, Colon, Embryo, Esophagus, Eye, Fibroblast, Heart, Kidney, Liver, Lung, Mesothelium, Nose, Oral, Other, Ovary, Pancreas, Prostate, Skin, Small intestine, Stomach, Testis, Thyroid |
| Cell species | Park-OxideNM2026 (10) | Chicken, Cow, Dog, Hamster, Human, Monkey, Mouse, Rabbit, Rat, Sheep |
|  | Park-OxideNM2026-HQ (7) | Chicken, Hamster, Human, Monkey, Mouse, Rat, Sheep |
|  | Park-OxideNM2026-Top4 (10) | Chicken, Cow, Dog, Hamster, Human, Monkey, Mouse, Rabbit, Rat, Sheep |
